# Additional Improvement of Respiratory Technique on Vascular Function in Hypertensive Postmenopausal Women Following Yoga or Stretching Video Classes: The YOGINI Study

**DOI:** 10.3389/fphys.2020.00898

**Published:** 2020-08-27

**Authors:** Cláudia Fetter, Juliana Romeu Marques, Liliane Appratto de Souza, Daniela Ravizzoni Dartora, Bruna Eibel, Liliana Fortini Cavalheiro Boll, Sílvia Noll Goldmeier, Danielle Dias, Katia De Angelis, Maria Cláudia Irigoyen

**Affiliations:** ^1^Clinical Investigation Laboratory (LIC), Cardiology Institute of Rio Grande do Sul/Cardiology University Foundation (IC-FUC), Porto Alegre, Brazil; ^2^Sainte Justine Hospital and Research Center, Montreal, QC, Canada; ^3^Department of Physiology, Federal University of São Paulo (UNIFESP), São Paulo, Brazil; ^4^Laboratory of Translational Physiology, Universidade Nove de Julho (UNINOVE), São Paulo, Brazil; ^5^Experimental Laboratory of Hypertension, Heart Institute (InCor), University of São Paulo (USP), São Paulo, Brazil

**Keywords:** hypertension, arterial stiffness, endothelial function, yoga, breathing, oxidative stress

## Abstract

**Background:** Hypertension remains highly prevalent in postmenopausal women, along with vascular dysfunction and increased oxidative stress. In such context, regular exercises, yoga practice, and slow breathing have been recommended to treat hypertension. However, the effects of the multiple components of yoga, including the respiratory techniques involved in the practice, on hypertension and on vascular and endothelial function have never been evaluated.

**Objective:** This study aimed to investigate the additional effects of respiratory technique on vascular function and oxidative stress profile in hypertensive postmenopausal women (HPMWs) following yoga or stretching video classes.

**Study Design:** Hypertensive postmenopausal women were recruited and randomized for 12 weeks, twice a week, of supervised yoga or stretching video classes of 75 min for 12 weeks associated or not with respiratory technique. Baseline and post-intervention measurements included pulse wave velocity (PWV), flow-mediated dilation (FMD), and oxidative stress parameters. Hypertensive postmenopausal women (59 ± 0.7 years) who ended the protocol were distributed into three groups: (1) control group (yoga or stretching, C, *n* = 14); (2) yoga + respiratory technique (Y+, *n* = 10); (3) stretching + respiratory technique (S+, *n* = 9).

**Results:** Diastolic blood pressure and FMD [baseline: C: 6.94 ± 1.97%, Y+: 7.05 ± 1.65%, and S+: 3.54 ± 2.01% vs. post: C: 16.59 ± 3.46% (*p* = 0.006), Y+: 13.72 ± 2.81% (*p* = 0.005), and S+: 11.79 ± 0.99% (*p* = 0.0001)] have significantly increased in all groups when baseline and post-practice values were compared. However, resting heart rate and PWV [baseline: Y+: 10.44 ± 3.69 and S+: 9.50 ± 0.53 m/s vs. post: Y+: 9.45 ± 0.39 (*p* = 0.003) and S+: 8.02 ± 0.47 m/s (*p* = 0.003)] decreased significantly only in the Y+ and S+ groups (baseline vs. post). Systemic antioxidant enzyme activities (superoxide dismutase and catalase) increased in all groups, and hydrogen peroxide and lipoperoxidation reduced in Y+ and S+ (baseline vs. post).

**Conclusions:** Twelve weeks of yoga or stretching video classes promoted positive changes in several outcomes generally regarded as cardiovascular risk factors in HPMWs, and these changes were even more pronounced by the association with respiratory technique.

## Introduction

There is a remarkable increased prevalence of hypertension in postmenopausal women (Yanes and Reckelhoff, 2011; Modena, 2014). Hypoestrogenism caused by menopause exerts deleterious effects on several tissues and organs, including vessels (Somani et al., 2019). This may lead to endothelial dysfunction (Sanchez-Barajas et al., 2018) arterial stiffness (Muka et al., 2016; Costa-Hong et al., 2018), and unfavorable oxidative stress profile (Dinh et al., 2014).

The mechanisms by which menopause leads to changes in vascular bed and impairment in endothelial function are complex and diverse, including decreasing vasodilation capacity and impairing the ability of signaling of blood flow, endothelium, and smooth muscle cells of the media layer (Somani et al., 2019). This process improperly generates reactive oxygen species (ROS), causing losses for vascular homeostasis (Hsieh et al., 2014). Along with this, increasing vascular resistance may represent a burden for hypertension and an overload for central arteries (Thijssen et al., 2016). Damage caused by mechanic stress of blood flow on walls of central arteries leads to lower compliance and arterial stiffness (Mitchell, 2009; Laurent, 2012). Arterial stiffness, in turn, increases with age, but the deleterious effects of menopause should not be seen as part of the natural aging process (Costa-Hong et al., 2018).

Taken together, these conditions demand appropriate clinical management, which should include non-pharmacological strategies in order to prevent their progression to cardiovascular diseases (Yanes and Reckelhoff, 2011). Among others, regular physical exercises and slow breathing are highly recommended to treat the effects of hypertension (Cornelissen and Smart, 2013), and in recent years, yoga also has been found to be an effective intervention (Hagins et al., 2013). Despite controversial results of yoga on arterial stiffness (Patil et al., 2015, 2017), an association between poor trunk flexibility and arterial stiffness has already been demonstrated (Yamamoto et al., 2009). Regarding endothelial function, several studies have demonstrated the effectiveness of yoga (Hunter et al., 2017, 2018). However, the impact of slow breathing on endothelial and vascular function has not yet been demonstrated (Limberg et al., 2013; DeLucia et al., 2018). Multiple components of yoga such as physical poses (*asanas*), respiratory exercises (*pranayamas*), meditation, and devotional and lifestyle aspects have never been analyzed separately in order to assess specific benefits (Hartley et al., 2014).

*Pranayamas* present different forms and speeds of inhalation, exhalation, and retentions (Jerath et al., 2006). *Ujjayi* is a slow-breathing *pranayama*, which narrows the glottis and extends respiratory phases. It decreases respiratory rate, and it is usually performed along with physical poses (Satin et al., 2014). Physical poses take limbs and spine to great range of motion, demanding mostly agonists' isometric contractions and antagonists' muscle group stretching (Jorge et al., 2016). In recent years, stretching has been regarded as an exercise able to promote changes on vascular function (Kato et al., 2017). Positive effects of single bouts of stretching have been reported, although mechanisms involved in such responses are not fully elucidated, and no studies have been carried out on chronic effects of this kind of exercise (Kruse et al., 2016; Kruse and Scheuermann, 2017).

As an innovative investigation, we may well hypothesize that in hypertensive postmenopausal women (HPMWs) additional improvements resulting from yoga poses and respiratory technique may be expected for blood pressure, vascular, and endothelial function, and oxidative stress profile after 12 weeks of video classes twice a week. Therefore, the aim of this study was to investigate the additional effect of respiratory techniques on vascular function and oxidative stress profile in HPMWs, following 12 weeks of yoga or stretching video classes.

## Methods

### Ethical Considerations

The Ethical Committee of Instituto de Cardiologia do Rio Grande do Sul/Fundação Universitária de Cardiologia approved this study, which is in accordance with CONSORT statement. All participants signed an informed written consent form. Data have been collected between July 2018 and December 2019. The study was registered in the Clinical Trials Registry (NCT03137849). All data have been fed into the REDCap Platform (www.redcap.cardiology.org.br).

### Participants

Patients have been recruited from the institution patient data, along with a social media network (i.e., Facebook). Inclusion criteria were as follows: age 45 to 68 years old, minimum of 12 months of amenorrhea, follicle-stimulating hormone >35 mUI/mL, blood pressure >140/90, or in continuous use of medication [diuretics, Ca^+^ channel inhibitors, angiotensin-converting enzyme (ACE) inhibitors, ARA2], not undergoing hormone replacement therapy, leading a sedentary lifestyle, and no previous yoga practice. Exclusion criteria were as follows: use of β-blockers and/or psychiatric medication, recent cardiovascular events or surgery, renal alterations, respiratory, and/or neuromotor pathologies, smoking, and body mass index >34.9 kg/m^2^.

### Randomization and Outcome Measures

After signing the informed consent form, participants were randomized and underwent baseline assessments at Clinical Investigation Laboratory and Ergometry Room of Institute of Cardiology of Rio Grande do Sul/Cardiology University Foundation by trained personnel blinded to the randomization. They were randomized into yoga, stretching, yoga + respiratory technique, and stretching + respiratory technique interventions. Given the dropout of subjects, and the lack of differences between yoga and stretching in baseline and post-interventions in the assessed parameters, participants were assigned into three groups: (1) control (yoga or stretching interventions alone, C, *n* = 14); (2) yoga + respiratory technique (Y+, *n* = 10), and (3) stretching + respiratory technique (S+, *n* = 9).

The participants were previously advised to fast overnight (12 h) and to refrain from alcohol, caffeine, and intensive exercise practice and were told to have proper night of sleep 24 h prior the examination day.

On the day of the evaluation, anthropometric measures, and body composition by bioimpedance were taken (BIODYNAMICS 310™). Systolic (SBP), diastolic blood pressure (DBP), and heart rate were assessed according to the American Health Association guideline recommendations. Blood sample were collected for biochemical analysis and systemic oxidative stress evaluations.

Arterial stiffness and endothelial function were assessed as described in the following section. Exercise electrocardiogram was performed to rule out any cardiac disease and to estimate maximal oxygen uptake (VO_2max_). Flexibility was evaluated with “Sit and Reach” test (Ayala et al., 2012).

After baseline assessments, participants attended 12 weeks (24 classes) of intervention and underwent final evaluations in the same baseline order. All participants have been told they were attending a yoga protocol. Blind investigators have taken all outcomes assessments to the interventions.

All evaluations have been repeated in the same order after completion of protocol, as follows:

### Biochemical Analyses

Fasting blood samples were collected to analyze fasting glucose (automated enzymatic method). Total cholesterol, high-density lipoprotein cholesterol, and triglycerides were assessed by the enzymatic colorimetric method. Follicle-stimulating hormone was assessed by the electrochemiluminescence method.

### Arterial Stiffness

Arterial stiffness was assessed by pulse wave velocity (PWV), which refers to the time a systolic wave travels in an arterial segment (Costa-Hong et al., 2018). Augmentation Index (Aix) refers to the sum of both anterograde systolic wave and the previously reflected systolic wave, and it is considered a reliable measure for vascular resistance along the arterial tree (Mitchell, 2009). Arterial stiffness was assessed by Complior Analyzer™ (ALAM Medical, France). Sensors were positioned upon the right carotid and femoral arteries. Distance between carotid and femoral pulse was provided by the investigator. Measures of blood pressure, height, and weight were fed into the software. Three consecutive measures were obtained from the equipment at a quality signal >90%. The mean of these three measures was considered for analysis of PWV, Aix, and central SBP and DBP (cSBP and cDBP, respectively) (Townsend et al., 2015).

### Endothelial Function (Flow-Mediated Dilation)

A high-resolution ultrasonography equipment (Esaote My Lab 70X Vision) was used for the evaluation of endothelial function through a high-frequency transducer to obtain longitudinal images of the brachial artery. The transducer was positioned upon the brachial artery in the 1/3 arm size of superior antecubital fossa. Baseline images were recorded for 1 min, and this was immediately followed by a cuffing inflated up to 200 mmHg and kept for 5 min in order to characterize reactive hyperemia. Thirty seconds before the cuffing was released, new images started to be recorded for 3 min, considered endothelium-dependent dilation, and were analyzed by the Cardiovascular Suite™ software (Quipu, Italy). The software demands to specify the interest area of the arterial segment and flow using visual selection. Baseline and post-hyperemia diameter and flow were computed to obtain the percentage of dilation, volume, and shear stress (Thijssen et al., 2019).

### Oxidative Stress Evaluations

#### Blood Sample Collection and Preparation

Whole blood was sampled from the participants in EDTA tubes and then centrifuged at 2,000 rotations per minute (rpm) during 10 min at 4°C. Plasma was removed and kept aside for further analysis. Mononuclear cell fraction was removed, and the red blood cells were washed with phosphate-buffered saline and centrifuged three times, for 5 min each, at 2,000 rpm. Further, in 100 μL of washed red blood cells, 1 mL of acetic acid (1 mM) and MgSO_4_ (4 mM) was centrifuged at 3,000 rpm for 30 min at 4°C. The supernatant was stored at −80°C for subsequent assessments. Proteins were quantified by the method described by Lowry et al. (1951).

#### Antioxidant Enzymes: Catalase and Superoxide Dismutase

Catalase (CAT) activity was evaluated by spectrophotometry (240 nm), through the consumption of hydrogen peroxide (H_2_O_2_; Sigma–Aldrich Corporation, H3410) by measuring decreasing absorbance, whose rate of decomposition is straightly proportional to its activity. Superoxide dismutase (SOD) activity was determined through measures of oxidative pirogalol (Sigma–Aldrich Corporation, P0381). A colorful by-product based on oxidation of pirogalol was detected by spectrophotometry (420 nm, SP22, Bioespectro) (Fridovich, 1986).

#### Nicotinamide Adenine Dinucleotide Phosphate Oxidase

Nicotinamide adenine dinucleotide phosphate oxidase (NADPH) oxidase was determined by the rate of NADPH consumption assessed by measuring the decline in absorbance (340 nm) every 10 min, using a plate reader spectrophotometer (Espectra Max 2, Molecular Devices) (Wei et al., 2006). For the assay, we used a 50 mM phosphate buffer containing EDTA (2 mM, Nuclear, 311737), sucrose (150 mM, Sigma–Aldrich Corporation, S7903), NADPH (1.3 mM, Sigma–Aldrich Corporation, N1630) and 10 μL of sample.

#### Hydrogen Peroxide (H_2_O_2_)

H_2_O_2_ was analyzed through measuring of oxidation of phenol red (Sigma–Aldrich Corporation, H3410) mediated by radish peroxidase (Sigma–Aldrich Corporation, P8250), using a plate reader spectrophotometer (610 nm, Espectra Max 2, Molecular Devices) (Pick and Keisari, 1980).

#### Lipoperoxidation

Plasma lipid peroxide levels were determined by measuring thiobarbituric acid reactive substances (TBARSs), a common method for measuring the concentration of malondialdehyde, the main breakdown product of oxidized lipids. For the TBARS assay, using 250 μL of sample, trichloroacetic acid (10%, wt/vol, Dinamica, 1072-1) was added to the homogenate to precipitate proteins and to acidify the samples. This mixture was then centrifuged (4,000 rpm, 10 min), the protein free sample was extracted, and thiobarbituric acid (0.67%, wt/vol, Sigma–Aldrich Corporation, T-550-0) was added to the reaction medium. The tubes were placed in a water bath (100°C) for 30 min. The absorbences were measured at 535 nm using a spectrophotometer (SP22, Bioespectro) (Buege and Aust, 1978).

#### Protein Oxidation

Carbonyls represent a marker of the oxidative damage to proteins and were assessed by the reaction of oxidative proteins in plasma with 2,4-dinitrophenylhydrazine (DNPH, Sigma–Aldrich Corporation, D199303) in acid mean. This was followed by successive washings with acid and organic solvents in the final incubation with guanidine hydrochloride solution (6M, Sigma–Aldrich Corporation, G4505). Absorbance of carbonyls was measured by spectrophotometry (360 nm, SP22, Bioespectro) (Reznick and Packer, 1994).

### Intervention

The 12 week supervised video classes occurred twice a week between 2:00 and 6:00 p.m and have taken place in a room equipped with a 32-inch-screen television and yoga mats.

Four video classes have been created as intervention: yoga, stretching, yoga + respiratory technique, and stretching + respiratory technique. The respiratory technique employed as intervention was *ujjayi pranayama* (victorious breath), a nasal respiration that narrows the glottis in order to extend both inspiratory and expiratory phases, and was performed along with yoga or stretching poses. Yoga and stretching, as control intervention, took only “inhale/exhale” commands. After the 60 min of yoga or stretching, all groups underwent the same yoga-based relaxation technique in supine position (15 min). The protocols were developed by same experienced yoga and stretching licensed instructor.

Yoga included three full sequences of sun salutations, followed by traditional standing poses, balance poses, stabilizations (core positions) and retroversion poses, sit poses, and final poses in supine position.

Stretching was based on dynamic (warm-up) and static exercises excluding those similar to yoga poses, attaining great range of motion of the main body joints and muscle groups, and did not include any body weight bearing, thus avoiding isometric contractions.

Full video classes of yoga and stretching were compiled as yoga (Videos 1–6) and stretching (Videos 7–12) for electronic version (in Portuguese audio, associated with respiratory technique).

A comparative plot of interventions is displayed in Supplemental Table 1.

### Data Analysis

The study was not analyzed as “intention to treat.” A secondary analysis of those participants who ended the protocol was provided. Because of losses in the follow-up, yoga and stretching not associated with respiratory technique were considered as one active control group, so statistical analyses were performed for three intervention groups: (1) control group (yoga or stretching), (2) yoga + respiratory technique (Y+), (3) stretching + respiratory technique (S+). Power of study was calculated *a posteriori* considering PWV variance among groups for post-intervention values, which was determined as β = 0.83. Medication classes used by participants were divided into five categories: (1) none, (2), diuretics, (3) angiotensin-converting enzyme inhibitor, (4) angiotensin II receptor blockers (ARBs), (5) combination of any class. This classification was taken to determine any differences among groups concerning the use of drug classes.

Collected data were processed by SPSS Statistics for Windows, version 25.0. Differences between post-intervention and baseline measures and among groups were calculated to determine changes in the outcome measures by GEE (generalized estimating equation), in order to obtain population-averaged effects. Bonferroni *post-hoc* test was performed. Two factors have been considered for analysis: intervention group, named “group,” and moment of evaluation (baseline and post-intervention), named “moment,” as well as interaction between them, called “interaction.” Data are shown as mean (M) ± standard error (SE). Correlations of Pearson for parametric data and Spearman (non-parametric) have been taken. Significance considered a *p* ≤ 0.05.

## Results

### Study Population

**Figure 1:**
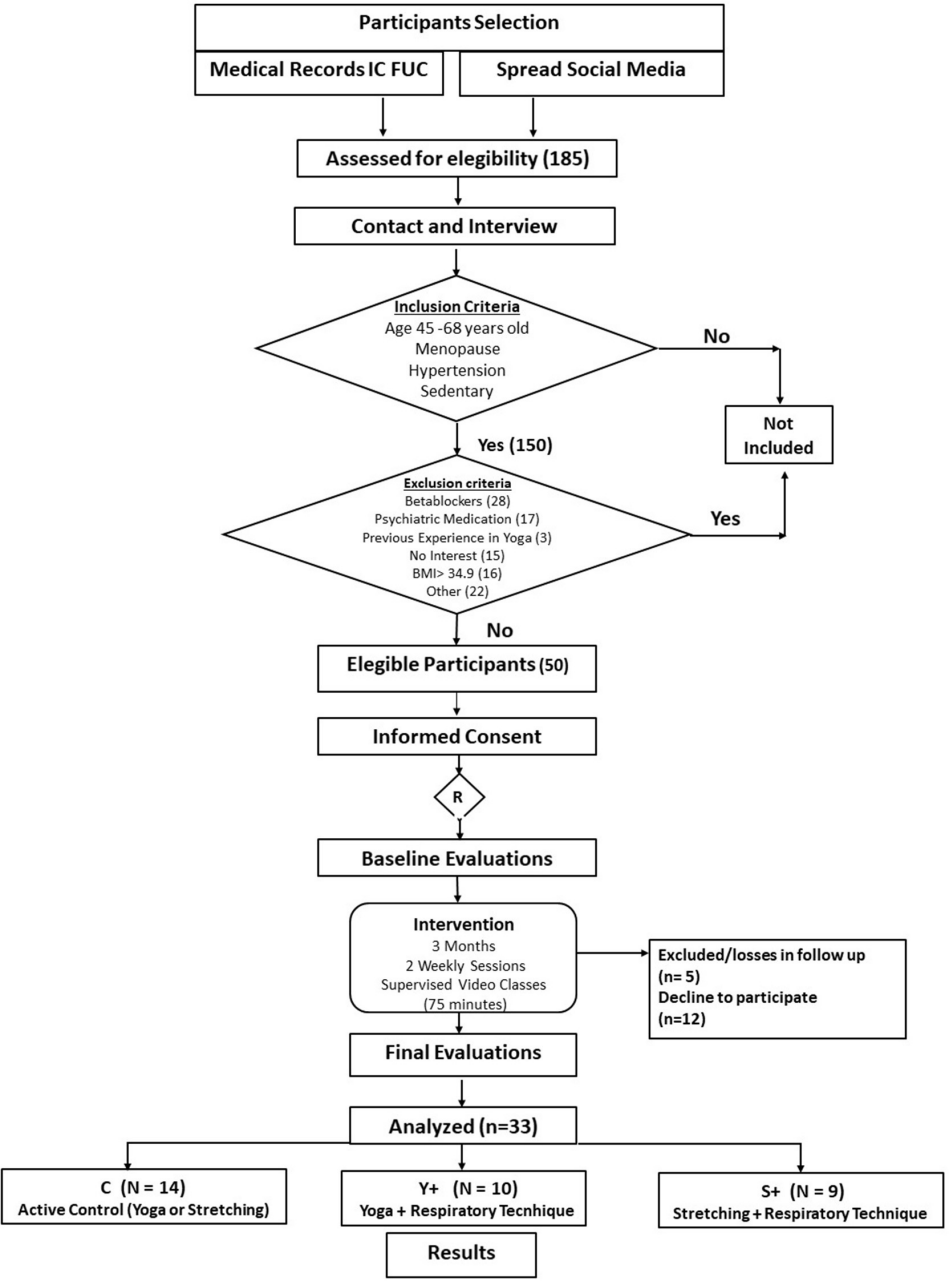
Flowchart of recruitment.

The eligible study population consisted of 50 women, of which 33 completed the 12 week protocol (Figure 1). Participants' age and time of menopause were similar among groups (Table 1).

**Table 1:** Characterization of participants after randomization, at baseline, and post-intervention for 12 weeks of video classes two times a week.

	**C (*n* = 14)**		**Y + (*n* = 10)**		**S + (*n* = 9)**		**GEE**		
	**Baseline**	**Post**	**Baseline**	**Post**	**Baseline**	**Post**	***P* group**	***P* moment**	***p* interaction**
Age (years)	59 ± 1,1	_	58.00 ± 1.2	_	60.44 ± 1.1	_			
Time of menopause (years)	9.5 ± 1.6	_	9.40 ± 1.54	_	10.22 ± 1.65	_			
**Anthropometric measures**									
BMI (kg/cm^2^)	27.68 ± 0.7	27.36 ± 0.7	25.93 ± 1.33	26.09 ± 1.33	29.43 ± 0.47	29.08 ± 0.50^*^	0.019	0.105	0.047
Fat Percentage (%)	35.66 ± 1.1	35.79 ± 1.16	34.34 ± 1.30	34.80 ± 1.44	38.94 ± 0.72	39.32 ± 0.63	0.001	0.233	0.984
Abdom circunference (cm)	85.25 ± 2.08	82.57 ± 2.38^*^	84.06 ± 3.15	82.39 ± 2.95	92.67 ± 1.55	88.83 ± 2.29^*^	0.019	0.001	0.601
**Biochemychal analyses**									
Glucose (mg/dL)	107.9 ± 5.3	103.6 ± 4.9	92.9 ± 2.6	96.4 ± 3.9	100.9 ± 6.8	100.7 ± 3.8	0.145	0.835	0.100
Total cholesterol (mg/dL)	217.3 ± 7.5	199.8 ± 7.9^*^	237.3 ± 11.0	227.6 ± 11.1^*^	192.8 ± 13.0	189.5 ± 12.1	0.037	<0.001	0.132
Cholesterol HDL (mg/dL)	53.6 ± 2.0	55.1 ± 3.0	63.5 ± 6.5	62.2 ± 5.9	64.1 ± 6.8	65.4 ± 6.5	0.176	0.658	0.655
Cholesterol LDL (mg/dL)	130.2 ± 7.5	114.6 ± 7.9^*^	148.1 ± 12.9	134.1 ± 11.5^*^	105.1 ± 14.3	97.8 ± 11.9	0.077	<0.001	0.552
Triglycerides (mg/dL)	167.5 ± 20.1	145.5 ± 15.5	124.2 ± 11.8	117.2 ± 12.9	117.8 ± 12.3	120.8 ± 16.6	0.110	0.302	0.474
FSH (mUI/mL)	71.5 ± 5.4	67.7 ± 5.9	82.5 ± 11.3	85.1 ± 9.9	66.3 ± 9.2	64.6 ± 9.0	0.374	0.585	0.429
**Functional measures**									
VO_2_max (ml/kg/min)	30.1 ± 1.7	31.4 ± 1.6	31.85 ± 2.2	33.89 ± 2.3	27.6 ± 1.4	29.5 ± 1.9	0.167	0.110	0.927
Flexibility test (cm)	23.9 ± 2.0	29.1 ± 2.5^*^	21.6 ± 3.2	30.6 ± 3.1^*^	19.6 ± 3.0	24.9 ± 2.4^*^	0.388	<0.001	0.410
**Hemodynamic measures**									
SBP (mmHg)	142.9 ± 5.8	134.5 ± 4.1^*^	137.4 ± 3.8	136.9 ± 4.0	141.2 ± 4.7	125.6 ± 5.0^*^	0.70	<0.001	<0.001
DBP (mmHg)	82.66 ± 2.7	78.66 ± 2.5^*^	86.23 ± 2.18	83.10 ± 2.35^*^	87.15 ± 2.98	76.37 ± 3.01^*^	0.418	<0.001	0.015
Heart rate (bpm)	67.7 ± 1.9	66.9 ± 2.1	68.7 ± 2.6	63.7 ± 2.7^*^	66.4 ± 2.2	63.4 ± 2.0^*^	0.683	0.005	0.269
Respiratory rate (cpm)	15.5 ± 0.9	14.7 ± 0.9	14.2 ± 0.9	13.9 ± 0.9	16.5 ± 1.8	15.9 ± 0.9	0.145	0.090	0.807

*Data shown as mean (M) ± standard error (SE). C, control group (yoga poses or stretching); Y+, yoga poses plus respiratory technique group; S+, stretching plus respiratory technique group; BMI, body mass index; HDL, high-density lipoprotein; LDL, low-density lipoprotein; FSH, follicle-stimulating hormone; VO_2_max, maximal oxygen uptake; SBP, systolic blood pressure; DPB, diastolic blood pressure*.

* *Intragroup analyses, p ≤ 0.05 baseline vs. post by GEE*.

Five participants were not taking any medication, three participants used only diuretics, 4 participants used ACE inhibitors, nine participants used ARBs, and 12 used a combination of drugs, including three who used of calcium-channel blockers. There were no differences between treated and untreated participants and the type of drugs used in all groups.

Body mass index was not changed significantly in any group from baseline to post-intervention moments. However, abdominal circumference was significantly decreased when baseline and post-intervention were compared. This is displayed in Table 1, which shows a significant decrease in the C (*p* = 0.031) and in S+ groups (*p* = 0.009), but not in the Y+ group (*p* = 0.294).

### Biochemical Analysis

Total cholesterol levels and low-density lipoprotein (LDL) cholesterol decreased significantly in the C and Y+ groups when baseline and post-intervention were compared (*p* = 0.009, and *p* = 0.039). S+ did not present significant changes in these parameters. Other biochemical assessments were similar among groups and time of evaluation (Table 1).

### Functional Measures

Estimated maximal oxygen consumption was not changed in any group after intervention. Significantly increased flexibility was noticed in all groups through flexibility test (C: *p* = 0.034, Y+: *p* = 0.0001, and S+: *p* = 0.001; Table 1).

### Hemodynamic Measures

**Figure 2:**
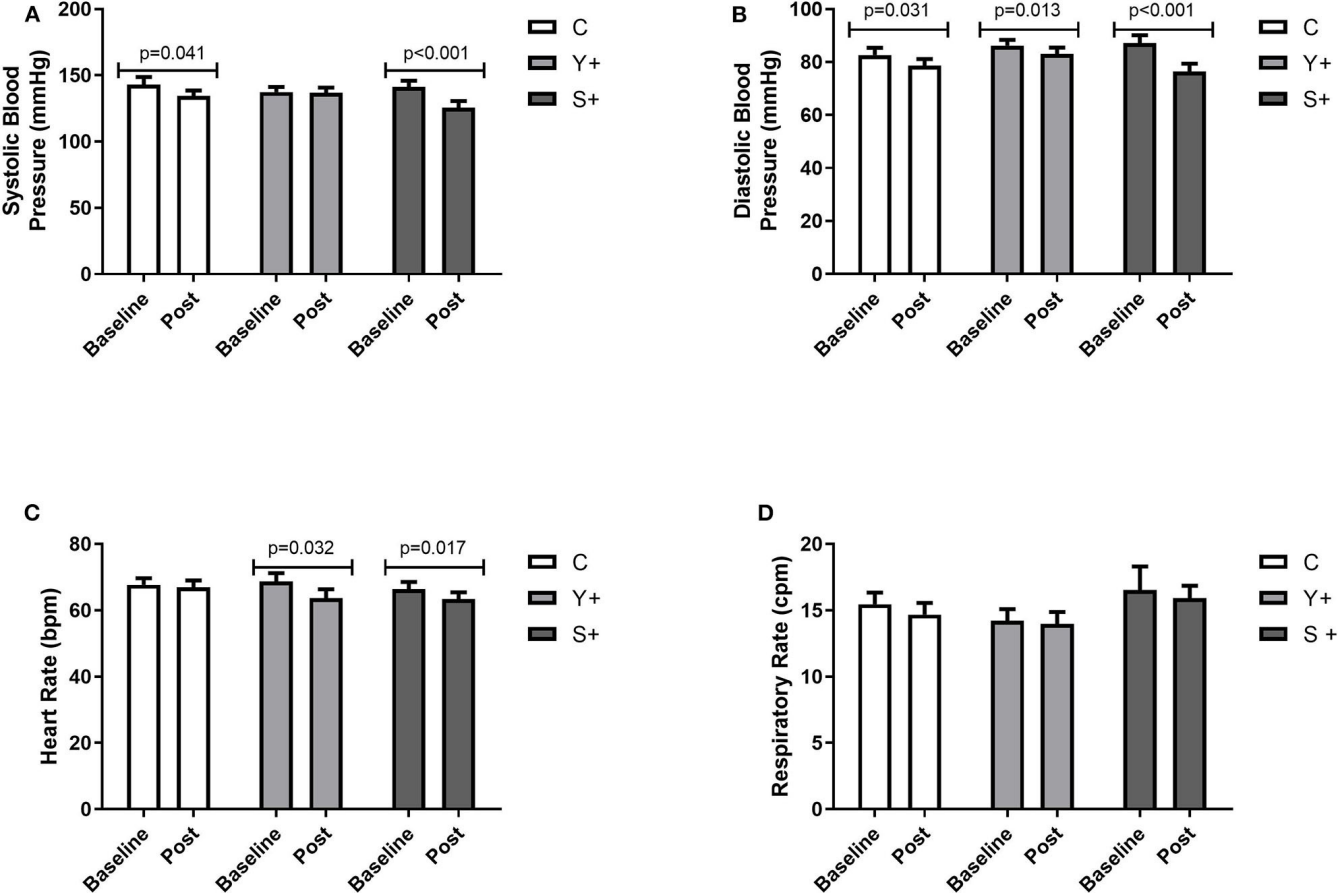
Hemodynamic and respiratory measures. **(A)** Systolic blood pressure expressed in mmHg. **(B)** Diastolic blood pressure expressed in mmHg. **(C)** Heart rate at rest expressed in beats per minute (bpm). **(D)** Respiratory rate expressed in cycles per minute (cpm). Baseline and post-intervention variations by generalized estimation equation (GEE) determined as Mean ± standard error. C, control group (yoga poses or stretching); Y+, yoga poses plus stretching technique group; S+, stretching plus control technique group.

This study has demonstrated a significant improvement from baseline to post-intervention values concerning hemodynamic measures, as demonstrated in Table 1 (moment baseline vs. post by GEE). Systolic blood pressure was decreased significantly in both the C and S+ groups (Figure 2A), whereas DBP decreased significantly in all groups (Figure 2B). Heart rates at rest decreased significantly in the Y+ and S+ groups, whereas the C group has not changed significantly when baseline and post-intervention were compared (Figure 2C). Respiratory rate was not changed significantly in any group (Figure 2D).

### Arterial Stiffness

**Figure 3:**
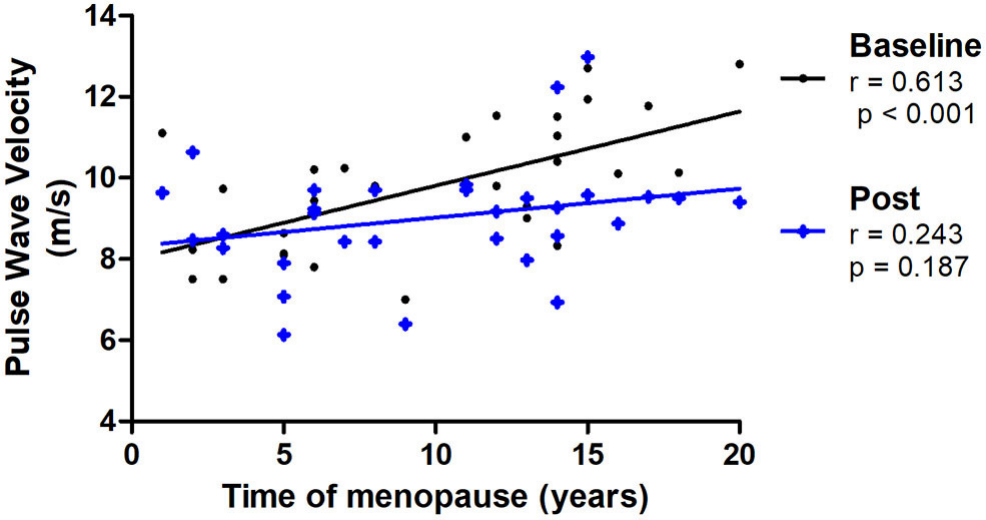
Correlations of PWV (pulse wave velocity) and time of menopause at baseline and post-intervention (*N* = 31).

**Figure 4:**
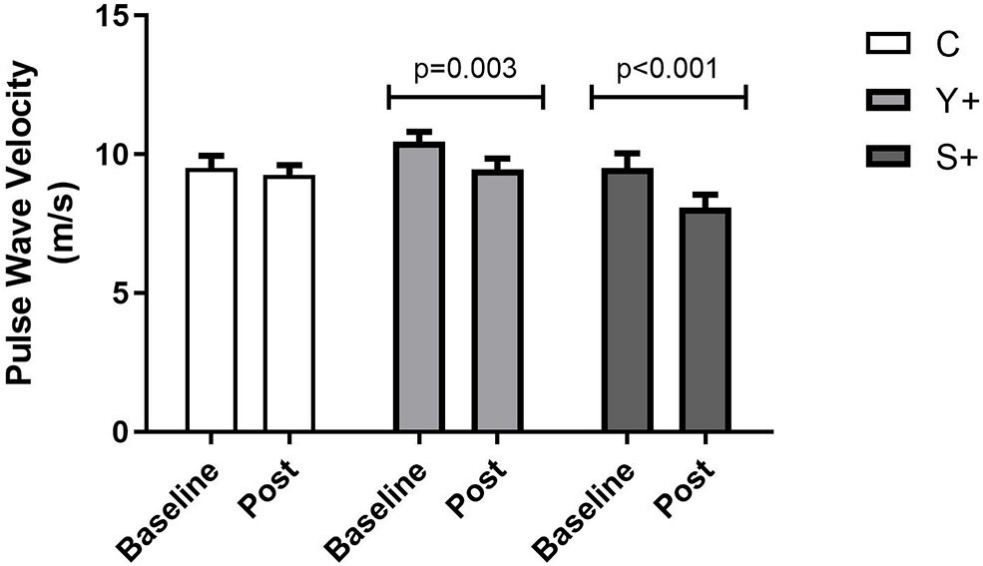
Arterial Stiffness assessed by pulse wave velocity (PWV) by Complior. Baseline and post-intervention variations determined by generalized estimation equation (GEE) determined as mean ± standard error. C, control group (yoga poses or stretching); Y+, yoga poses plus respiratory technique group; S+, stretching plus respiratory technique group.

Pulse wave velocity and years of menopause showed a moderate significant correlation at baseline (*r* = 0.613, *p* < 0.001), which was non-significant in the post-intervention assessment (*r* = 0.243, *p* = 0.187; Figure 3). Most outcomes of arterial stiffness demonstrated a significant improvement when baseline and post-intervention measurements were compared, as demonstrated in Table 2 (moment baseline vs. post by GEE). Augmentation Index was significantly decreased in the C group, and central DBP decreased in all groups when baseline and post-intervention were compared. The PWV decreased significantly only in both groups with respiratory technique post-intervention, but not in the control group (Figure 4).

**Table 2:** Vascular function-arterial stiffness and endothelial function [flow-mediated dilation (FMD)].

	**C (*n* = 14)**		**Y + (*n* = 9)**		**S + (*n* = 8)**		**GEE**		
	**Baseline**	**Post**	**Baseline**	**Post**	**Baseline**	**Post**	***P* group**	***P* moment**	***p* interaction**
**Arterial stiffness**									
Pulse wave velocity (m/s)	9.5 ± 0.4	9.3 ± 0.4	10.4 ± 0.4	9.5 ± 0.4*	9.5 ± 0.5	8.1 ± 0.5*	0.132	<0.001	0.076
Augmentation Index (%)	32.9 ± 4.2	22.7 ± 4.3*	29.2 ± 4.8	29.6 ± 3.4	39.9 ± 7.7	28.7 ± 2.9	0.525	0.032	0.221
cSBP (mmHg)	124.0 ± 4.3	119.8 ± 5.2	125.3 ± 3.9	123.0 ± 3.2	125.5 ± 5.9	119.2 ± 5.7	0.893	0.058	0.681
cDBP (mmHg)	79.8 ± 2.7	76.5 ± 2.7*	87.9 ± 2.8	83.2 ± 2.1*	87.3 ± 2.8	79.5 ± 3.2*	0.085	<0.001	0.293
**Endothelial function**									
Rest diameter (mm)	4.05 ± 0.17	3.89 ± 0.16	3.95 ± 0.26	3.87 ± 0.20	4.00 ± 0.24	3.83 ± 0.23	0.963	0.331	0.973
FMD diameter (mm)	4.30 ± 0.15	4.50 ± 017	4.22 ± 0.28	4.42 ± 0.30	4.13 ± 0.24	4.29 ± 0.28	0.767	0.291	0.988
FMD (%)	6.94 ± 1.97	16.59 ± 3.46*	7.05 ± 1.65	13.72 ± 2.81*	3.54 ± 2.01	11.79 ± 0.99*	0.187	<0.001	0.764
TP (s)	64 ± 14	63 ± 12	42 ± 11	83.0 ± 16.	83 ± 21	75 ± 15	0.388	0.450	0.250
Rest mean flow (ml)	129 ± 23	103 ± 17	83 ± 15	133 ± 16*	64 ± 22	104 ± 18	0.332	0.106	0.022
Mean flow FMD (ml)	316 ± 32	304 ± 45	393 ± 47	455 ± 61	250 ± 53	341 ± 75	0.036	0.268	0.501
Rest mean shear stress (dynes/cm^2^)	170 ± 34	164 ± 29	126 ± 26	216 ± 38*	137 ± 61	206 ± 57	0.994	0.022	0.116
Mean shear stress FMD (dynes/cm^2^)	381 ± 44	324 ± 36	555 ± 79	571 ± 63	436 ± 119	498 ± 64	0.001	0.907	0.641

*Arterial stiffness assessed by Complior™ and flow-mediated dilation (FMD) at baseline and post-intervention for 12 weeks of video classes two times a week. Data shown as mean (M) ± standard error (SE). C, control group (yoga poses or stretching); Y+, yoga poses plus respiratory technique group; S+, stretching plus respiratory technique group. Pulse wave velocity (m/s), Augmentation Index (%), cSBP, central systolic blood pressure (mmHg); cDBP, central diastolic blood pressure (mmHg); FMD, flow-mediated dilation; TP, time to peak*.

### Endothelial Function

**Figure 5:**
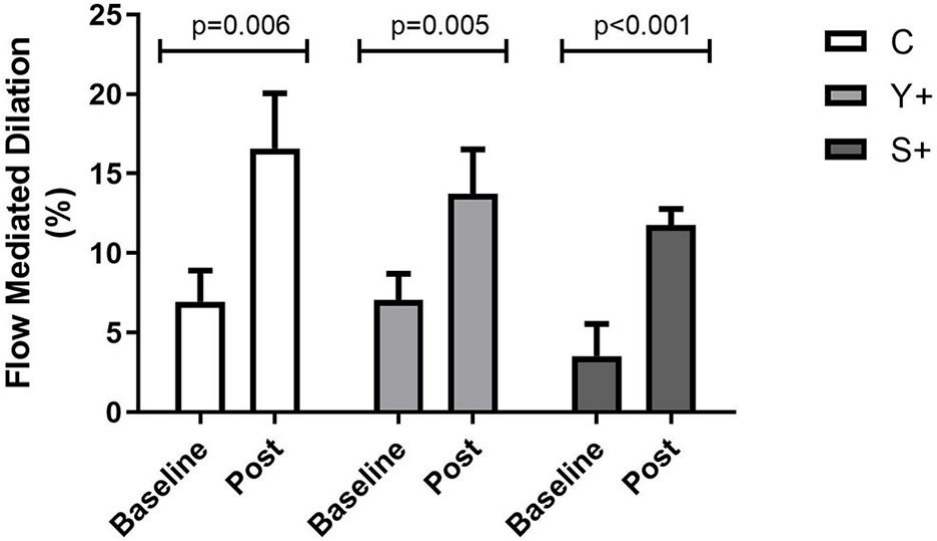
Endothelial function by flow-mediated dilation (FMD) at baseline and post-intervention variations determined by generalized estimation equation (GEE) determined as mean ± standard error. C, control group (Yoga poses or stretching); Y+, yoga poses plus respiratory technique group; S+, stretching plus respiratory technique group.

Rest value diameters were no changed significantly, but flow-mediated dilation (FMD) (%) had a significant increase in all groups after interventions (Figure 5). Time to peak (TP), mean flow of FMD, and mean shear stress of FMD did not present any significant change. However, rest mean flow and mean shear stress were increased significantly only in the Y+ group. The data are shown in Table 2.

### Oxidative Stress

All systemic oxidative stress parameters demonstrated significant improvement when baseline and post-intervention measurements were compared (moment baseline vs. post by GEE), as demonstrated in Table 3. Regarding antioxidants, SOD, and CAT activities showed significant increases in all intervention groups when baseline and post-intervention values were compared. Although NADPH oxidase was not different, H_2_O_2_ concentration and lipoperoxidation (TBARS) decreased significantly in the Y+ and S+ groups after interventions. Protein oxidation (carbonyls) showed significant decrease in C and Y+ groups.

**Table 3:** Oxidative stress.

	**C (*n* = 12)**		**Y+ (*n* = 9)**		**S + (*n* = 8)**		**GEE**		
	**Baseline**	**Post**	**Baseline**	**Post**	**Baseline**	**Post**	***P* group**	***P* moment**	***p* interaction**
SOD (USOD/mg protein)	5.15 ± 0.097	6.15 ± 0.13^*^	5.36 ± 0.12	6.11 ± 0.16^*^	5.01 ± 0.19	6.11 ± 0.18^*^	0.566	<0.001	0.533
CAT (nmol/mg protein)	2.17 ± 0.07	2.34 ± 0.06^*^	2.28 ± 0.11	2.59 ± 0.12^*^	2.00 ± 0.11	2.47 ± 0.13^*^	0.290	<0.001	0.229
NADPH (nmol/mg protein)	0.031 ± 0.003	0.026 ± 0.002	0.029 ± 0.003	0.026 ± 0.002	0.030 ± 0.004	0.027 ± 0.002	0.575	0.056	0.082
H_2_O_2_ (μM H_2_O_2_)	23.6 ± 2.38	21.7 ± 2.94	26.5 ± 3.41	18.6 ± 3.54^*^	32.9 ± 3.42	22.3 ± 3.83^*^	0.272	0.005	0.078
TBARS (μmol/mg protein)	0.41 ± 0.03	0.36 ± 0.03	0.38 ± 0.04	0.26 ± 0.03^*^	0.39 ± 0.04	0.27 ± 0.03^*^	0.281	0.001	0.885
Carbonyls (nmol/mg protein)	1.85 ± 0.13	1.43 ± 0.09^*^	1.86 ± 0.16	1.49 ± 0.12^*^	1.69 ± 0.05	1.51 ± 0.09	0.685	0.003	0.476

*Oxidative stress variables at baseline and post-intervention for 12 weeks of video classes two times a week. Data shown as mean (M) ± standard error (SE). C, control group (yoga poses or stretching); Y+, yoga poses plus respiratory technique group; S+, stretching plus respiratory technique group; SOD, superoxide dismutase; CAT, catalase; H_2_O_2_, hydrogen peroxide; NADPH, nicotinamide adenine dinucleotide phosphate oxidase; TBARS, thiobarbituric acid reactive substances*.

* *Intragroup analyses, p ≤ 0.05 baseline vs. post by GEE*.

## Discussion

This innovative investigative study was able to demonstrate multiple significant improvements in HPMWs after 12 weeks of yoga or stretching video classes, with additional effects of respiratory technique. General decreases in blood pressure concur to beneficial effects of these practices and are in accordance with previous findings for yoga (Hagins et al., 2014) and respiratory techniques (Pinheiro et al., 2007).

Along with decreased heart rate at rest, both groups with respiratory technique were able to significantly decrease PWV. Previous findings for yoga have demonstrated decreased PWV (Patil et al., 2015), but to our knowledge, this is the first study to demonstrate that protocols including respiratory technique may influence PWV in HPMWs.

Respiration and heart rhythms respond to their interrelated changes (Dick et al., 2014) and heart rate may modulate vascular endothelium, acting on mechanical pulsatile stress (Laosiripisan et al., 2017). Changes in intrathoracic and transmural pressure during inspiration influences cardiac filling and stroke volume, generating stroke volume variability (Shibata et al., 2006). Thus, changes in pattern of respiration such as expansibility of rib cage may influence hemodynamics. Responses of venous return and atrial filling to respiration may be associated to the pattern of respiration, instead of rate (Byeon et al., 2012). Increasing venous return due to diaphragmatic breathing has been demonstrated in healthy individuals (Miller et al., 2005; Balzan et al., 2014). We may speculate that the decreased PWV and rest heart rate in both groups with respiratory technique in the present study are, at least in part, mediated by these mechanisms.

A moderate significant correlation between PWV and years of menopause at baseline was noticed, as expected (Thijssen et al., 2016). At the end of interventions, this correlation disappeared, showing an attenuation of the effects of menopause on PWV, possibly caused by these non-pharmacological interventions.

Moreover, the prevention of losses in nitric oxide bioavailability should be a goal in HPMWs because estrogen deprivation reduces it and increases ROS (Green et al., 2014) thus increasing risk for atherosclerosis (Witkowski and Serviente, 2018). The increase in FMD in all intervention groups found in this study corroborates previous findings of increased FMD after Bikram yoga (hot yoga) (Hunter et al., 2018) and strengthens the potential role of this practice to prevent deleterious effects of menopause. Increased FMD has also been found after some types of exercise training (Early et al., 2017) including isometric exercises (Badrov et al., 2016) which are present in many yoga poses of this study protocol.

Accordingly, increased systemic antioxidant defenses were noticed in this study, once SOD and CAT, as a frontline expression of it, were increased in all groups. As a unique complex producing only ROS, the tendency to decrease (*p* = 0.056, moment baseline vs. post by GEE) in NADPH oxidase (an important source of superoxide anion) and the reduction in systemic hydrogen peroxide (H_2_O_2_) concentration in the Y+ and S+ groups indicate an overall decrease of pro oxidants. Regarding oxidative stress damage, lipoperoxidation (TBARS) was reduced when baseline and post-intervention values were compared in the Y+ and S+ groups. Moreover, carbonyls, as markers of oxidative damage to proteins were significantly decreased in groups undergoing yoga intervention (C and Y+, baseline vs. post). Taken together, our findings demonstrate markedly improvements in antioxidant, pro-oxidants, and biomolecule damage after interventions, suggesting additional effects in yoga groups and in the groups undergoing respiratory technique. In fact, beneficial effects of yoga on oxidative stress in elderly hypertensive subjects have been previously demonstrated (Patil et al., 2014) along with the beneficial effects of stretching on oxidative stress profile of heart failure patients (Sankaralingam et al., 2011; Kato et al., 2017).

Perhaps increasing fascicle length and local shear stress by ischemia during stretching, present in both yoga poses and stretching, may provide an overall reduction in peripheral resistance through reducing smooth muscle cells tone, besides other adaptations that may induce changes in endothelial function and oxidative stress profile (Wong and Figueroa, 2014; Kruse and Scheuermann, 2017).

Increased flexibility might be considered a functional achievement, once losses in flexibility are expected in postmenopausal women. Although there were no significant increases in estimated VO_2max_, decreases in total cholesterol and LDL in C and S+ point to a possible metabolic improvement.

The main limitation of this study lies in the relatively high number of dropouts−17 out of 50 recruited HPMWs—higher than other yoga studies (most carried out in developed countries), which reduced the number of subjects in each group. However, despite that, the power of study calculated *a posteriori*—considering PWV variance among groups for post-intervention—was β = 0.83.

In summary, our findings demonstrated that yoga poses and stretching supervised video classes for 12 weeks improved blood pressure, arterial stiffness, endothelial function, and oxidative stress profile in HPMWs. Effects of respiratory technique along with yoga poses or stretching point to possible improvement in arterial stiffness. Therefore, yoga or stretching, even when administered through video classes seems to have a positive impact on several outcomes regarded as cardiovascular risk factors, and these benefits are extended by the association of respiratory technique. Nevertheless, more research based on more robust yoga and stretching interventions and a larger sample are needed to lend further support to our findings.

## Data Availability Statement

All data supporting the conclusions of this study will be fully provided on request by authors.

## Ethics Statement

The studies involving human participants were reviewed and approved by Comitê de Ética do Instituto de Cardiologia - 5273/16 - 03/11/2016. The patients/participants provided their written informed consent to participate in this study.

## Author Contributions

All authors listed have made a substantial, direct and intellectual contribution to the work, and approved it for publication.

## Conflict of Interest

The authors declare that the research was conducted in the absence of any commercial or financial relationships that could be construed as a potential conflict of interest.
