# Supplementary figures and images for: Additional Improvement of Respiratory Technique on Vascular Function in Hypertensive Postmenopausal Women Following Yoga or Stretching Video Classes: The YOGINI Study
Source: Front Physiol. 2020 Aug 27;11:898. doi: 10.3389/fphys.2020.00898 (PMC7485134; doi:10.3389/fphys.2020.00898)

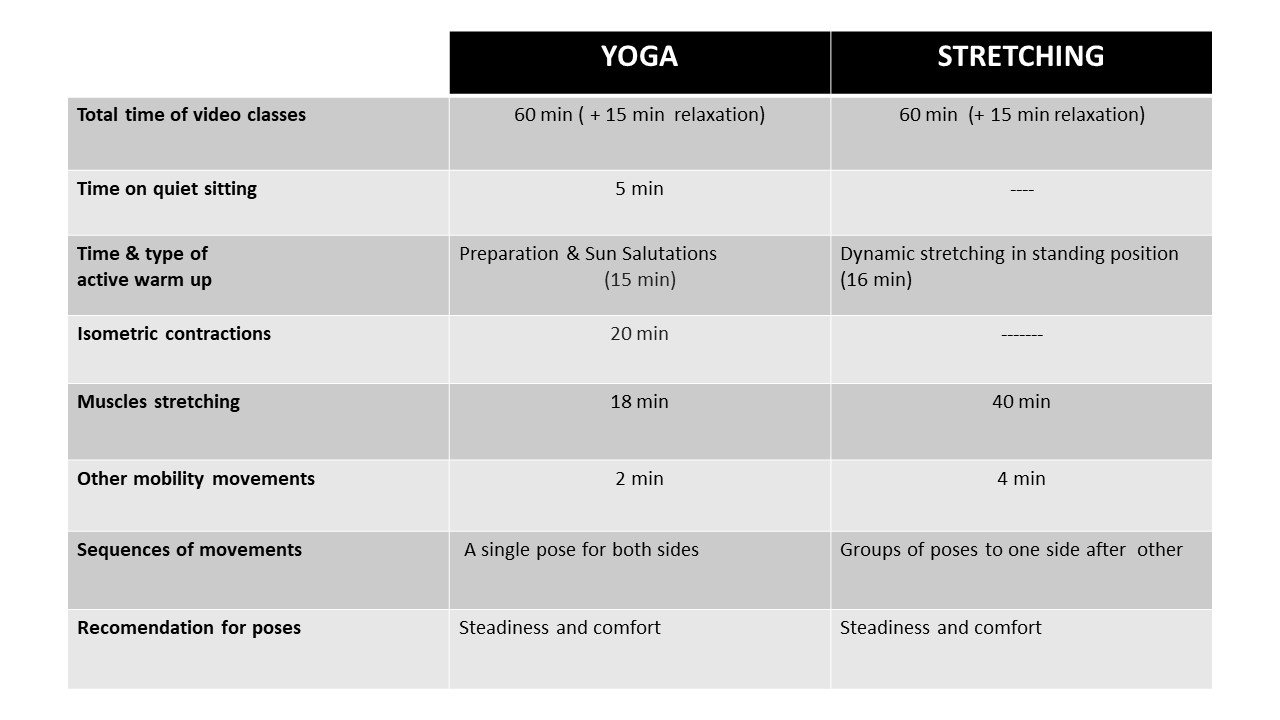

Supplement: Supplementary file 13 [file Image_1.jpg]
